# Bayesian maximum entropy-based prediction of the spatiotemporal risk of schistosomiasis in Anhui Province, China

**DOI:** 10.1186/s12879-021-06854-6

**Published:** 2021-11-22

**Authors:** Fuju Wang, Xin Liu, Robert Bergquist, Xiao Lv, Yang Liu, Fenghua Gao, Chengming Li, Zhijie Zhang

**Affiliations:** 1 College of Geomatics, Shandong University of Science and Technology, Qingdao, 266590 China; 2 School of Public Health, Fudan University, Shanghai, 200032 China; 3 Anhui Institute of Schisomiasis Control and Research, Hefei, 230061 China; 4 Chinese Academy of Surveying and Mapping, Beijing, 100036 China; 5 Geospatial Health, Ingerod, Brastad, Sweden

**Keywords:** Bayesian maximum entropy, Geographical and temporal weighted regression, Schistosomiasis, Spatiotemporal kriging, Spatiotemporal interpolation

## Abstract

**Background:**

“Schistosomiasis” is a highly recurrent parasitic disease that affects a wide range of areas and a large number of people worldwide. In China, schistosomiasis has seriously affected the life and safety of the people and restricted the economic development. Schistosomiasis is mainly distributed along the Yangtze River and in southern China. Anhui Province is located in the Yangtze River Basin of China, with dense water system, frequent floods and widespread distribution of *Oncomelania hupensis* that is the only intermediate host of schistosomiasis, a large number of cattle, sheep and other livestock, which makes it difficult to control schistosomiasis. It is of great significance to monitor and analyze spatiotemporal risk of schistosomiasis in Anhui Province, China. We compared and analyzed the optimal spatiotemporal interpolation model based on the data of schistosomiasis in Anhui Province, China and the spatiotemporal pattern of schistosomiasis risk was analyzed.

**Methods:**

In this study, the root-mean-square-error (RMSE) and absolute residual (AR) indicators were used to compare the accuracy of Bayesian maximum entropy (BME), spatiotemporal Kriging (STKriging) and geographical and temporal weighted regression (GTWR) models for predicting the spatiotemporal risk of schistosomiasis in Anhui Province, China.

**Results:**

The results showed that (1) daytime land surface temperature, mean minimum temperature, normalized difference vegetation index, soil moisture, soil bulk density and urbanization were significant factors affecting the risk of schistosomiasis; (2) the spatiotemporal distribution trends of schistosomiasis predicted by the three methods were basically consistent with the actual trends, but the prediction accuracy of BME was higher than that of STKriging and GTWR, indicating that BME predicted the prevalence of schistosomiasis more accurately; and (3) schistosomiasis in Anhui Province had a spatial autocorrelation within 20 km and a temporal correlation within 10 years when applying the optimal model BME.

**Conclusions:**

This study suggests that BME exhibited the highest interpolation accuracy among the three spatiotemporal interpolation methods, which could enhance the risk prediction model of infectious diseases thereby providing scientific support for government decision making.

## Background

Schistosomiasis, an important zoonotic parasitic disease caused by three main (and three less common) species of the trematode worm Schistosoma, is reported from 78 countries on the tropical and subtropical parts of the world where it affects more than 200 million people [1]. *Schistosomiasis japonicum* is endemic in China [2], where its endemic areas are classified into three types based on geographical topography and the ecological characteristics of breeding areas of the only intermediate snail host *Oncomelania*: lakes and swamp areas, plain areas of waterway networks, and hilly and mountainous areas [3, 4]. Compared with the latter two types of area, schistosomiasis control has proven difficult in the lake and swamp areas because of the widespread distribution of breeding areas and difficult-to-control water levels, where over 80% of schistosomiasis cases occur [5, 6]. Frequent flooding of the Yangtze River that runs through Anhui Province forming lakes and swamps adds to the problem. The large number of livestock, such as cattle and sheep that play the role of reservoir hosts in endemic areas, exacerbate the difficulty of controlling transmission of the disease facilitating the persistence of schistosomiasis in the country. This situation contributes to the great significance of the disease and the need to study its risk potential in Anhui Province [2].

Because of the large workload associated with schistosomiasis control, the number of surveillance areas varies from year to year. This causes results in incomplete and irregular schistosomiasis data that not only poses an obstacle for control efforts but also affects people’s judgment of the schistosomiasis risk potentially leading to unsafe and hazardous behaviour [7]. Data interpolation is the primary approach to solving the problem of missing spatiotemporal data regarding the risk of schistosomiasis. However, as traditional data interpolation methods tend to study temporal or spatial interpolation separately which makes a global view elusive. Commonly used spatial interpolation methods include inverse distance-weighted interpolation [8] and Kriging interpolation [9] to convert data from discrete points into a continuous data surface. From a spatiotemporal analysis point of view, however, one-sided spatial interpolation analyzes confines the analysis to a particular point or period of time which destroys the uniformity of the spatiotemporal continuum. On the other hand, temporal interpolation is to interpolate the observed time series; commonly used such methods include the autoregressive model [10], the autoregressive moving average model [11] and the generalized additive model [12]. Time series analysis of spatiotemporal data alone greatly reduces the pure spatial correlation. These shortcomings have led to spatiotemporal interpolation methods, which are widely used today for the estimation of missing spatiotemporal datasets, generating high-precision, spatiotemporal surfaces expressing spatiotemporal processes and distributions [3, 13, 14]. The main spatiotemporal interpolation methods are spatiotemporal Kriging (STKriging) [3, 13, 14] and regression-based methods [15–19], such as geographical and temporal weighted regression (GTWR) and Bayesian maximum entropy (BME). Both STKriging interpolation and GTWR interpolation have been applied to the study of schistosomiasis, the former of which has divides the prevalence of schistosomiasis into spatiotemporal trends and residuals [13], whereas the latter of which has used factors that affect schistosomiasis to fit the prevalence [20]. The spatiotemporal variation functions of the residuals are first established and the residuals of the prevalence of schistosomiasis are then predicted based on that, with the final interpolation results obtained by summing the spatiotemporal trends and the predicted residual. Considering the characteristics of the above two interpolation methods, this study attempts to improve the accuracy of predicting the risk of schistosomiasis by taking into account the factors influencing the prevalence value when attempting to predict it around the point to be estimated based on the values measured. This approach is the BME method [21–34], and it refers to high-order statistical estimation of the spatiotemporal prevalence phenomenon and predicts the risk of disease at the point to be estimated based on soft data (e.g., data fitted according to mathematical or statistical methods, uncertain, subjective or qualitative data) and hard data (data actually measured around the point to be estimated). The BME method has been successfully applied to infectious diseases such as syphilis [25], hand-foot-mouth disease [27], influenza [23, 38], dengue fever [30, 32] and Black Death [24]. However, it has been rarely been applied to the prediction of the risk of schistosomiasis.

The prevalence of schistosomiasis is interpolated in Anhui Province with BME in the study. The spatiotemporal pattern of schistosomiasis risk is analyzed based on interpolation results.

## Methods

### Study area

Anhui Province is located in southern China, with an area of approximately 140,100 km^2^ and had a population of approximately 636,570,000 in 2019. The province is crossed by the Huai River in the north and the Yangtze River in the south. Climatically, it is a transitional area between tropical and subtropical zones, with warm temperatures north of the Huai River and a subtropical zone south of the Huai River. There is a pronounced monsoon climate with strong rains in June and July, which often lead to flooding. Such geography and climate are well suited for the growth and reproduction of the schistosomiasis-related Oncomelania snails. Thus, Anhui Province is of key concern for control of this disease, and study therefore focused on the endemic lakes and swamps along the Yangtze River Basin as it traverses the province.

### Prevalence data

**Fig. 1:**
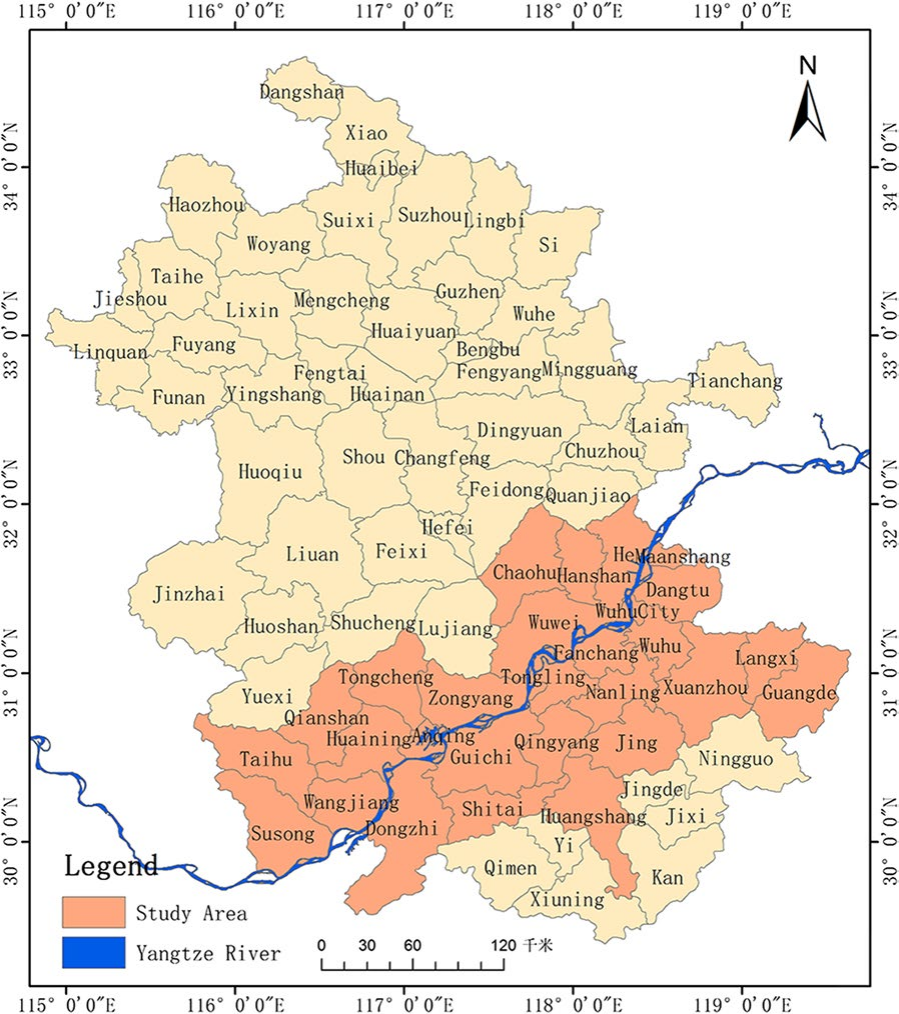
The study area. This figure was produced in ArcGIS 10.2(ESRI, Redlands, CA, USA) using shape files representing county-level administrative units in Anhui Province freely downloaded from Resource and Environment Science and Data Center (http://www.resdc.cn/data.aspx?DATAID=201)

Data on the prevalence of schistosomiasis infection in Anhui Province between 2000 and 2015 were obtained from field surveys conducted by professional health workers at the Anhui Institute of Parasitic Diseases (AIPD) [3]. A two-step diagnostic approach was used annually to identify cases of schistosomiasis infection: serology was done for all people aged 5 to 65 years in endemic villages using the indirect hemagglutination test (IHT) followed by faecal Kato-Katz parasitological test for those with positive blood test results [35]. The results were reported to the AIPD through the county office [3]. The study covered 29 counties between 2000 and 2015 (Fig. 1).

### Environmental data

As transmission of schistosomiasis [13] is closely related to the presence of Oncomelania intermediate host snails in the natural environment as well as social factors, such as urbanization, which combine to influence the spatiotemporal distribution of schistosomiasis risk. In the present study, daytime land surface temperature (LSTd), night-time land surface temperature (LSTn), the normalized difference vegetation index (NDVI), meteorological data (precipitation, mean minimum temperature (MTmin), mean maximum temperature (MTmax) and sunshine hours), soil data (soil moisture, soil pH and soil bulk density), distance from the Yangtze River and the urbanization level (using night-time light data to describe the county-level urbanization level [36]) were chosen as the main factors influencing schistosomiasis presence.

LSTd, LSTn and NDVI data were obtained from the Level-1 and Atmosphere Archive & Distribution System (LAADS) website (https://ladsweb.modaps.eosdis.nasa.gov/), with a temporal resolution of 1 month and a spatial resolution of 1 km. ENVI Software (version 5.2, Research System Inc. (RSI), Boulder, CO, USA) was used for cropping and stitching the above data. The LSTd, LSTn and NDVI data of each county are pixel accumulation, respectively. Then, the monthly pixel average value of each county is calculated by using the zoning statistical function of ArcGIS software (version 10.4, ESRI Inc., Redlands, CA, USA), to produce county attribute tables. Finally, the annual average value of each county is calculated**.** Meteorological data, including precipitation, MTmin, MTmax and sunshine hours, came from the website of the China Meteorological Administration (http://data.cma.cn/) with a time resolution of one month. Meteorological data for Anhui Province with a spatial grid of 1 km × 1 km were obtained through Kriging interpolation. The monthly averages for each county were calculated using ArcGIS zoning statistics followed by production attribute tables and calculation of the annual averages for each county.

Soil moisture data were obtained from the European Space Agency (https://www.esa-soilmoisture-cci.org/) with a temporal resolution of 1 day and a spatial resolution of 0.25°. Soil pH and soil bulk density data were obtained from the Cold and Arid Region Scientific Data Center (http://westdc.westgis.ac.cn/data), with a temporal resolution of 1 year and spatial resolution of 30 arc-seconds. The obtained data were dealt as described above for the LSTd, LSTn, NDVI and the meteorological data.

Data regarding the distance to the Yangtze River came from the World Wildlife Fund (https://www.worldwildlife.org/) and the Euclidean distance measurement tool in ArcGIS software were used to calculate the distance from the geometry center to the Yangtze River in each county.

The night-time light data included the Defense Meteorological Program Operational Line-Scan System (DMSP-OLS) with its unique capability to detect visible and near-infrared light emission and the Visible Infrared Imaging Radiometer Suite (*VIIRS*), both instruments operated by the US National Oceanic and Atmospheric Administration (NOAA) (http://ngdc.noaa.gov/eog/download.html). We used DMSP-OLS data with a spatial resolution of 1 km, a temporal resolution of 1 year, and a time range of 1992–2013, where transient light, such as lightning and natural gas flares, had been removed. The National Polar-Orbiting Partnership's Visible Infrared Imaging Radiometer Suite (NPP-VIIRS) data had a spatial resolution of 500 m, a temporal resolution of 1 month, and a time range of 2000–2015. The noise in the NPP-VIIRS data was first removed, after which the night-time light index was calculated for each county; finally, the NPP-VIIRS night-time light index was converted to the DMSP-OLS night-time light index using a curve fitting method [36].

### Statistical analysis

A univariate analysis of the influencing factor data with the prevalence of schistosomiasis was performed first to exclude variables with *P* > 0.1[13]; then, a multicollinearity test was conducted on the remaining variables with the variance inflation factor (VIF) index < 5; finally, backward stepwise regression modelling was carried out with *P* > 0.1 and *P* ≤ 0.05 as the exit and entry criteria, respectively [13]. When modelling, the data were randomly divided into ten equal parts, nine of which were used as training data; the remaining part was used as the test validation data. The BME, STKriging, and GTWR interpolation analyses were performed to select the optimal interpolation model for analysis of the spatiotemporal patterns of schistosomiasis (see the Appendix for a description of the three methods). Statistical analyses (univariate analysis, multicollinearity test and backward stepwise regression) were carried out using the statistical software SPSS version20. BME computations were performed with the software SEKS-GUI v1.0.8 [22]. GTWR computation were carried using the software ArcGIS version10.4. STKriging were implemented in the R package gstat [37]

The RMSE and absolute residual (AR) of the validation datasets were used for comparison of the prediction accuracy of the different methods, assessing the prediction accuracy of the model over the whole study area, as well as in each county.

1 $$RMSE = \sqrt {\frac{1}{16n}\sum\limits_{{t_{i} = 2000}}^{2015} {\sum\limits_{i = 1}^{n} {y\left( {u_{i} ,v_{i} ,t_{i} } \right) - \hat{y}\left( {u_{i} ,v_{i} ,t_{i} } \right)^{{2}} } } }$$

2 $$AR = \left| {y\left( {u_{i} ,v_{i} ,t_{i} } \right) - \hat{y}\left( {u_{i} ,v_{i} ,t_{i} } \right)} \right|$$

where $(u_{i} ,v_{i} ,t_{i} )$ are the spatiotemporal coordinates of the geometric centre of the $i$-th county; $u_{i}$,$v_{i}$, and $t_{i}$ the longitude, latitude and time coordinates of the $i$-th sample point, respectively; $y(u_{i} ,v_{i} ,t_{i} )$, and $\hat{y}(u_{i} ,v_{i} ,t_{i} )$ the observed and predicted values of the prevalence of schistosomiasis in the $i$-th county in Anhui Province, respectively; and $n$ the number of counties in the province where schistosomiasis still is endemic.

ArcGIS software is used to analyze the temporal and spatial changes of the RMSE and AR.

## Results

### Analysis of influencing factors and projected results

Table 1 shows the results of the backward stepwise regression significance test on the data of influencing factors of schistosomiasis in Anhui Province between 2000 and 2015. The LSTd, MTmin, NDVI, soil moisture, night-time light, and soil bulk density were included in the model; their VIF values were all less than 5, and their P-values less than 0.05. This indicates that the collinearity between these influencing factors is small and that there is a significant relationship between these factors and the prevalence of schistosomiasis in the province.

**Table 1:** Significance test results of influencing factors

Influencing factor	*P*	VIF
LSTd	0.001	1.164
MTmin	0.041	1.034
NDVI	0.003	1.217
Soil moisture	0.0005	1.190
Night-time light data	0.034	1.033
Soil bulk density	0.000014	1.091

The GTWR model was then used to fit the significant influencing factors with regard to the prevalence of schistosomiasis. The goodness-of-fit R^2^ = 0.76 indicates that the GTWR model can reveal 76% of the spatiotemporal variation in the prevalence of schistosomiasis.

**Fig. 2:**
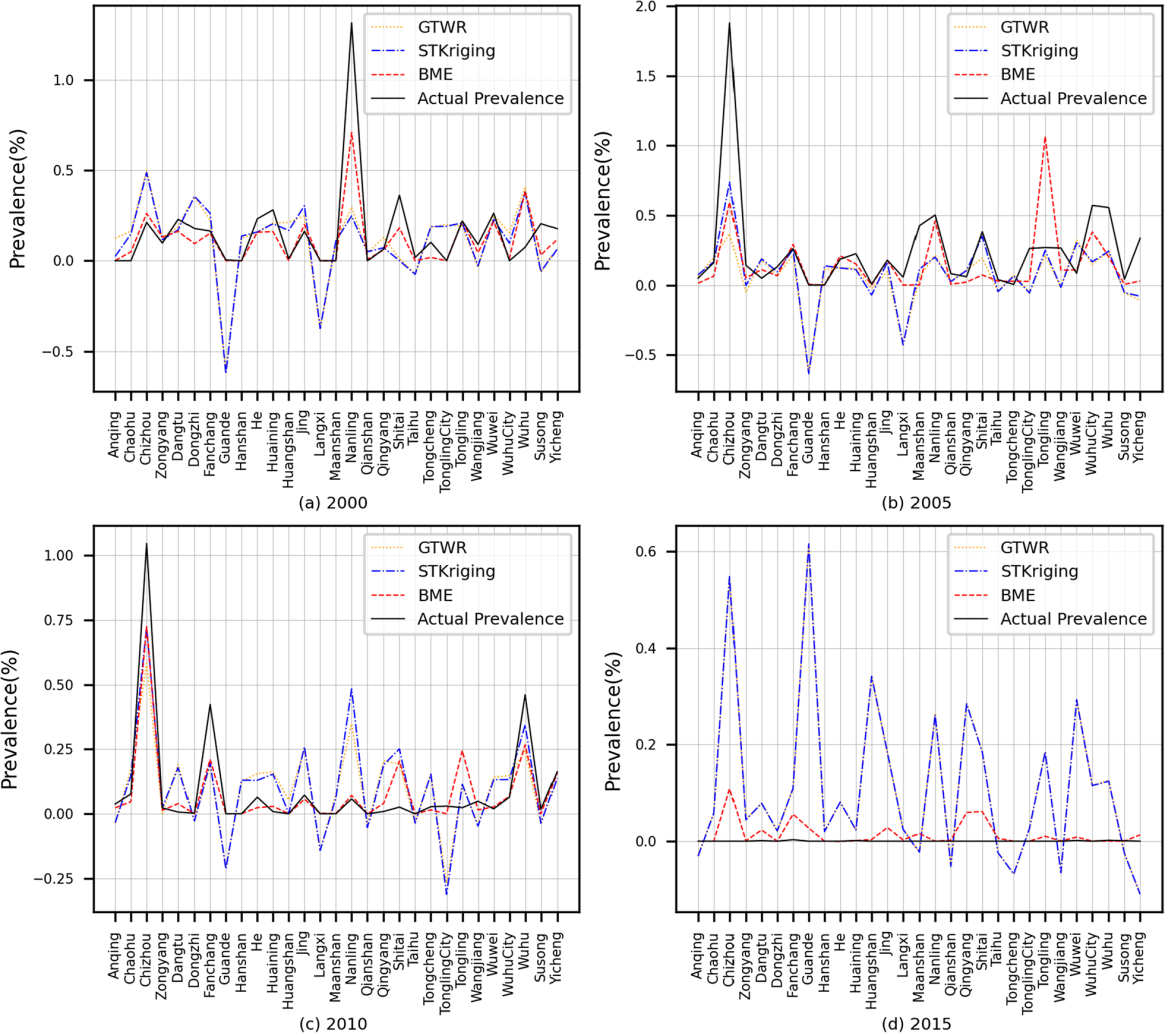
Model projections for the years 2000, 2005, 2010 and 2015

Figure 2 shows a comparison between the predicted and observed values of the three different interpolation methods for the years 2000, 2005, 2010 and 2015. The trends between the predicted and actual values of the three methods were basically consistent. The BME predictions showed a better fit at the maximum and minimum prevalence. In contrast, STKriging and GTWR interpolation results showed a poor fit at the maximum and minimum prevalence and the prediction results were poorer for years with lower prevalence, such as 2015 (e.g., these data were mostly 0).

### Comparison of prediction accuracy

**Fig. 3:**
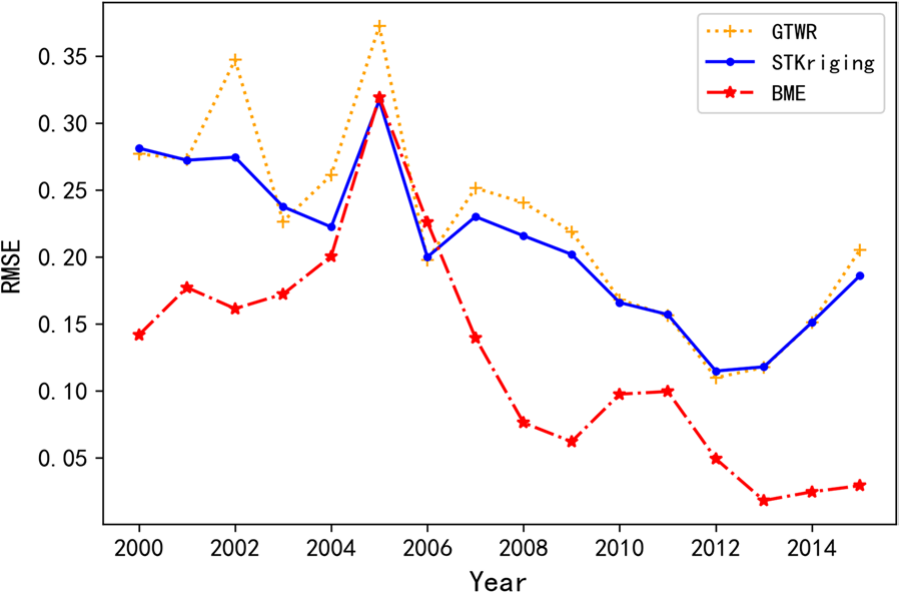
Comparison of the prediction accuracies of the three methods in the years covered by the study

The RMSE value of the ten-fold cross-validation of BME was 0.148, which was 0.071 and 0.087 lower than that of the STKriging (RMSE = 0.219) and GTWR (RMSE = 0.235), respectively. This suggests that the interpolation accuracy of BME is better than that of STKriging and GTWR. Figure 3 shows the RMSE comparison of the three interpolation methods for the annual prevalence of schistosomiasis in the 29 counties in Anhui Province. Overall, the RMSE of the BME interpolation method was lower than that of STKriging and GTWR, and the RMSE of STKriging was lower than that of GTWR in most years; however, these values were similar to each other.

**Fig. 4:**
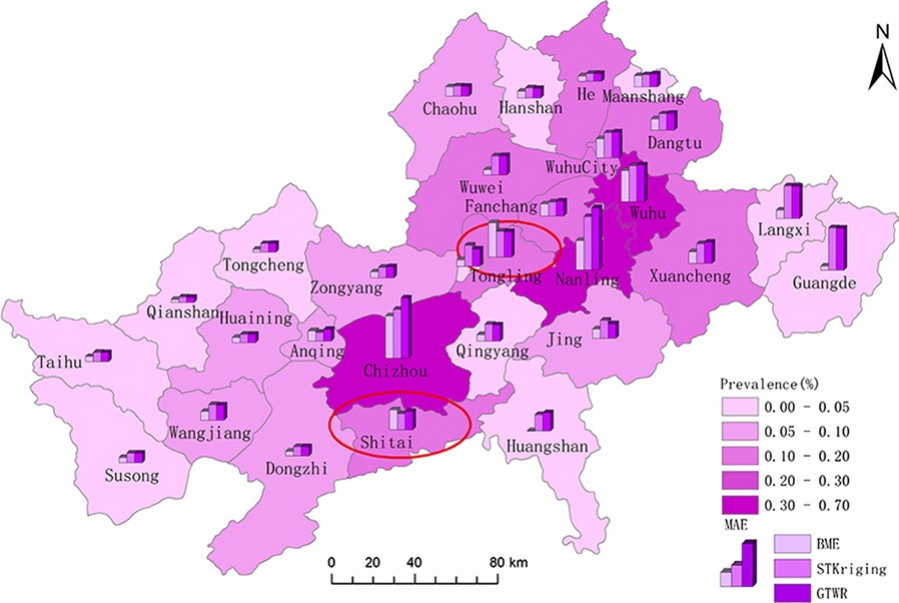
Comparison of prediction accuracies of the three methods in different regions of the Yangtze River Basin as it traverses Anhui Province. This figure was produced in ArcGIS 10.2 (ESRI, Redlands, CA, USA) using shape files representing endemic areas county-level administrative units in Anhui Province freely downloaded from Resource and Environment Science and Data Center (http://www.resdc.cn/data.aspx?DATAID=201)

Figure 4 shows a comparison of the RMSEs of the three methods for the prevalence of schistosomiasis in the 29 counties in Anhui Province. With the exception of counties Shitai and Tongling, the RMSEs of BME interpolation were lower than those of STKriging and GTWR. Meanwhile, the RMSEs of the STKriging interpolation method were slightly lower than those of the GTWR model in most districts and counties.

**Fig. 5:**
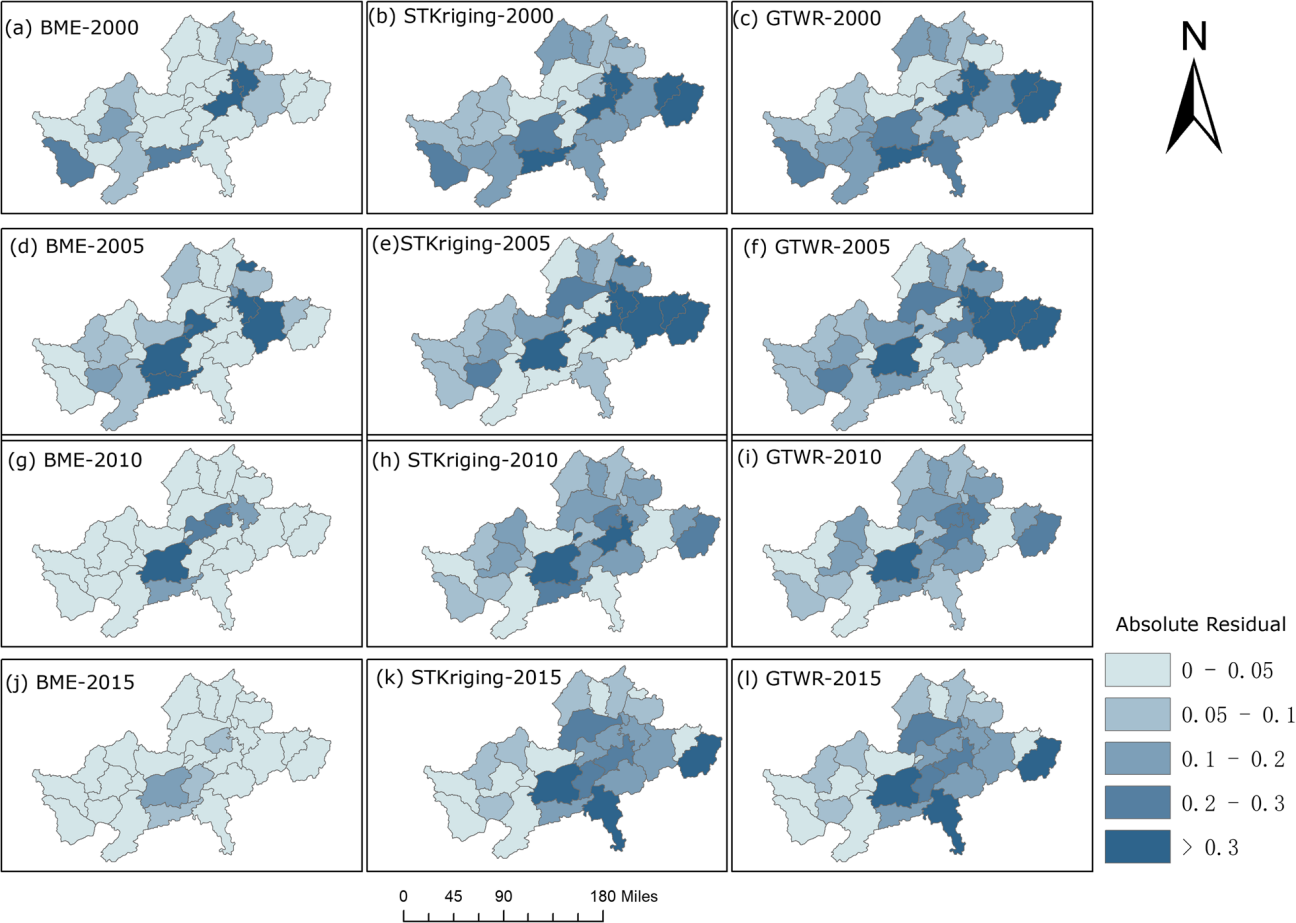
Spatial distribution of AR in the years 2000, 2005, 2010 and 2015. This figure was produced in ArcGIS 10.2 (ESRI, Redlands, CA, USA) using shape files representing endemic areas county-level administrative units in Anhui Province freely downloaded from Resource and Environment Science and Data Center (http://www.resdc.cn/data.aspx?DATAID=201)

To investigate the spatial interpolation accuracy of the three models, the errors of the three models were compared for the years 2000, 2005, 2010, and 2015 (Fig. 5). Overall, the BME model showed larger errors in the surrounding areas, such as Chizhou County and Shitai County. However, in terms of spatial distribution, the interpolation accuracy of BME was overall higher than that of STKriging and GTWR, whose spatial distributions of error were similar.

### Analysis of the spatiotemporal patterns of schistosomiasis prevalence in Anhui Province

**Fig. 6:**
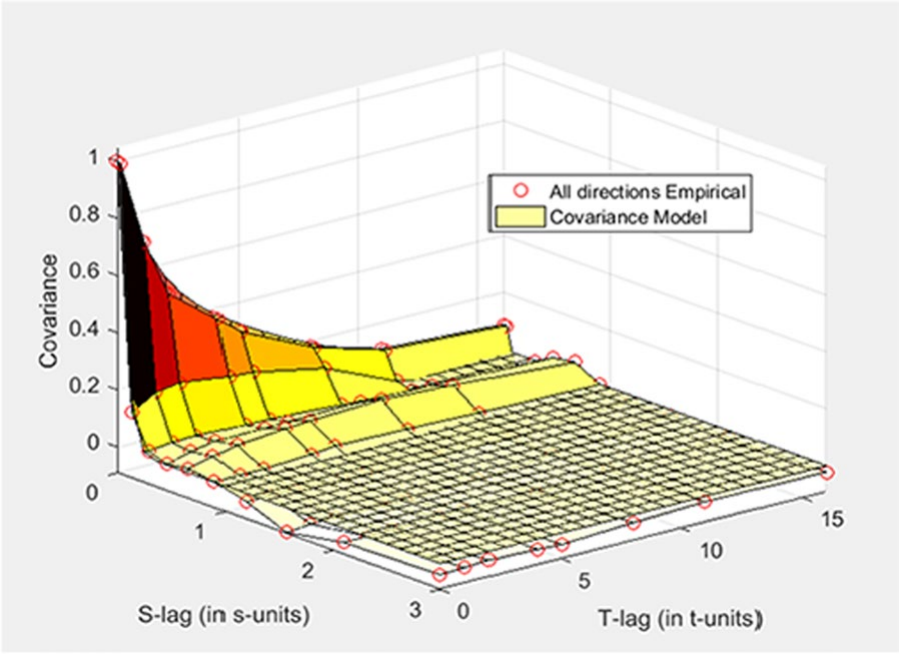
Spatiotemporal covariance of schistosomiasis prevalence using the BME method. S-lag refers to the space step(km), and T-lag refers to the time step(year)

The BME method showed the highest interpolation accuracy and was therefore used to examine the spatiotemporal patterns of schistosomiasis in Anhui Province. In this study, the temporal and spatial components of the combined exponential and spherical models were used to fit the spatiotemporal covariance of schistosomiasis prevalence as shown in Fig. 6. The spatiotemporal covariance model is expressed by Eq. (5) in the Appendix, where $c_{1}$ = 0.55, $c_{2}$ = 0.45, $a_{{h_{s} 1}}$ = 0.1 (ca. 10 km), $a_{{h_{t} 1}}$ = 17 years, $a_{{h_{s} 2}}$ = 0.3 (ca. 30 km), and $a_{{h_{t} 2}}$ = 4 years. A larger covariance value indicates a stronger spatiotemporal correlation of schistosomiasis. The figure shows that larger values of the spatiotemporal covariance occur within a spatial range of 0.20° (ca. 20 km) and a time range of 10 years.

## Discussion

We first investigated and identified the significant influencing factors closely related to schistosomiasis in Anhui Province. We then used the prevalence predicted by the GTWR model as soft data and the measured prevalence in the field as hard data for BME interpolation.

As an introduction of the use of the BME method for the prediction of the prevalence of schistosomiasis, we compared BME with the STKriging and GTWR methods for spatiotemporal interpolation in Anhui Province, China. The GTWR model established the regression relationship between the influencing factors and the prevalence of schistosomiasis; the STKriging model took the predicted values of the GTWR model as spatiotemporal trends and interpolated the residuals; and the BME model used for the GTWR predicted values as soft data together with the actual measured prevalence data as hard data for interpolation.

Overall, the interpolation accuracy of BME was higher than that of STKriging and GTWR, with the accuracy of STKriging slightly higher than that of GTWR. The STKriging model divided the prevalence of schistosomiasis into two components, the spatiotemporal trend and the residuals. The former was expressed by the GTWR and the residuals, for which the trend was not available, by the STKriging interpolation, assuming that the residuals satisfied second-order stationarity. The STKriging model incorporated more information on schistosomiasis and was thus more accurate for prediction than the GTWR model. The BME, using soft and hard data during interpolation, where the former were the GTWR fitted data and the latter the prevalence data that had been measured in the field, proved superior to both the other approaches. Importantly, the data did not need to meet the second-order stationarity and could automatically fit the nonlinear estimator [38, 39].

Spatially, all three models showed lower prediction accuracy in areas with high prevalence. Areas susceptible to the Yangtze River water level represent environments difficult to control. Examples of such places include Chizhou County, Shitai County, Nanling County and Wuhu County characterized by a multitude of rivers, lakes and beaches and the Oncomelania snails therefore widely distributed. The poverty alleviation policy in China have regrettably led to the development of a large snail areas results in some places with an increase schistosome-infected cattle and sheep acting as reservoirs of the disease. Consequently, the prevalence of schistosomiasis increases, with the complex influencing factors leading to a lower prediction accuracy. Only the BME model showed high prediction accuracy also in areas with low prevalence, such as Tongcheng County, Qianshan County, Taihu County, Susong County, Dongzhi County, Huangshan County, Langxi County, and Guangde County. Because of the low prevalence of schistosomiasis in these areas, there were continuous changes in significant influencing factors, leading to a high accuracy of BME soft data calculated based on the GTWR model.

From the temporal point of view, the interpolation results of all three models were basically consistent with the trends of actual prevalence measured, but the interpolation results of STKriging and GTWR models were poor after 2012. The prevalence of schistosomiasis in Anhui Province decreased appreciably after 2012, whereas the natural environment did not show significant changes. The factors influencing the prevalence of schistosomiasis are complex and varied, and it was difficult to simulate the spatiotemporal trend of schistosomiasis using natural and social factors alone. This, surely influenced the GTWR interpolation results negatively after 2012. The STKriging interpolation consisted of the spatiotemporal trend and residuals, and the interpolation results of this model agreed with those of GTWR, indicating that the interpolation accuracy of STKriging was primarily determined by the fitting accuracy of the spatiotemporal trend.

The parameters of the spatiotemporal covariance of the schistosomiasis infection rate characterized the spatial and temporal variations of the disease. Larger values of spatiotemporal covariance emerged within the spatial range of 0.20° (ca. 20 km). This revealed that there was spatial autocorrelation within 20 km and negligible spatial correlation beyond 20 km in Anhui Province, suggesting that schistosomiasis transmission was within districts and counties. In addition, the influence of the time scale was as long as 10 years, and the temporal correlation was negligible beyond 10 years, suggesting that the prevalence of schistosomiasis in endemic areas at the current low epidemic level could be autocorrelations and the correlation could be up to 10 years. This result essentially agrees with our previous findings [13].

Although the spatiotemporal distribution of schistosomiasis predicted by BME, STKriging, and GTWR in Anhui Province agrees with the observed spatiotemporal distribution of schistosomiasis, a discrepancy between the predicted and observed values of schistosomiasis remained (Fig. 2). The spread of schistosomiasis usually depends on factors that cause non-linear and rapid changes in schistosomiasis, whereas such changes are often not characterized by geostatistical models (e.g., GTWR), thus affecting the prediction accuracy of BME and STKriging. Figure 2 shows the highest prevalence and lowest interpolation accuracy in 2005. Possible reasons for this are the following two developments: (1) as the government initiated the World Bank Loan Project (1992–2001) for schistosomiasis control in Anhui province, the number of Oncomelania snails, showed a declining trend between 2001 and 2004. As the 10-year project was replaced in 2005 by an integrated control strategy more focused on infectious source control, some increase of Oncomelania snails may have started [2, 40]; and (2) the Yangtze River Basin had a warm winter and early spring in 2004 and 2005 followed by high humidity in the spring and summer leading to increased snail breeding and reproduction of Oncomelania snails [41]. These two changes were difficult to characterize using the GTWR model, reducing the prediction accuracy and further affecting the prediction accuracy of BME and STKriging. We have previously studied the impact of the schistosomiasis control project in Anhui province [41, 42], and our future priority will be to study the impact of nonlinear and rapidly changing factors on the risk of schistosomiasis.

## Conclusion

This study suggests that BME exhibited the highest interpolation accuracy among the mainstream spatiotemporal interpolation methods, which could enhance the risk prediction model of infectious diseases thereby providing scientific support for government decision making. The concluding findings were the following:

1. Urbanization together with five factors characterizing the environment in the rural areas influenced the prevalence of schistosomiasis at the county level in Anhui Province. The goodness of fit between these influencing factors and the schistosomiasis prevalence using GTWR was R^2^ = 0.76, which means that the influencing factors could explain 76% of the spatiotemporal distribution of schistosomiasis.
2. The prediction accuracy of BME was better what STKriging and GTWR could provide. Moreover, the predicted values all agreed with the actual spatiotemporal distribution trend of schistosomiasis.
3. Schistosomiasis outbreaks occurred in more than 20 counties per year in Anhui Province between 2000 and 2012, with the spatial range gradually decreasing after 2012. Schistosomiasis spread up to 20 km from the original areas and affected the province for up to 10 years, which may be related to the low level of disease intensity and testing results.

## Data Availability

LSTd, LSTn and NDVI data used in the study are available from LAADS (https://ladsweb.modaps.eosdis.nasa.gov/). Soil moisture data used in the study are available from the European Space Agency (https://www.esa-soilmoisture-cci.org/). Soil pH and soil bulk density data used in the study are available from the Cold and Arid Region Scientific Data Center (http://westdc.westgis.ac.cn/data). Data regarding the distance to the Yangtze River used in the study are available from the World Wildlife Fund (https://www.worldwildlife.org/). The night-time light data used in the study are available from NOAA(http://ngdc.noaa.gov/eog/download.html). For the other data, please contact the authors for a link to the raw data.

## Appendix

### Bayesian maximum entropy, BME

BME, initially proposed as a theoretical framework by Christakos in 1990, proves to be a method of nonlinear interpolation with higher precision. BME, used for spatiotemporal heterogeneity research, is designed to integrate data of various precision and quality which are called the Knowledge Base, KB. KB includes General Knowledge Base, G-KB and Specific Knowledge Base, S-KB. G-KB is used to describe the integral character of S/TRF, which can be signified as the statistical law such as expectation, variance as well as covariance. The S-KB includes hard data and soft data. Hard data are those with negligible errors, such as measured point data, while soft data are relatively imprecise data. The processes of BME are as follows:

(1) Calculating prior probability density function (PDF). According to the maximization principle of expected value of information (entropy), priori PDF with the distribution of unobserved variables can be calculated in the research area by utilizing G-KB.

$\chi_{map} = \left( {\chi_{hard,} \chi_{soft,} \chi_{k} } \right)$ is spatio-temporal random variable, and its priori PDF is represented by $f_{G} \left( {\chi_{map} } \right)$. Therefore, based on the principle of information entropy, the expectation of maximum entropy is

3 $$E\left[ {Info\left( {\chi_{map} } \right)} \right] = - \int {\ln } \left[ {f_{G} \left( {\chi_{map} } \right)} \right]f_{G} \left( {\chi_{map} } \right)d\chi_{map}$$

where $\chi_{hard,} \,\chi_{soft,} \,\chi_{k}$ respectively indicate hard data (annual measured morbidity of Schistosomiasis in 29 epidemic counties in Anhui Province from 2000 to 2015), soft data (morbidity of Schistosomiasis measured annually in 29 epidemic counties in Anhui Province from 2000 to 2015 according to calculation of GTWR model) and morbidity in the point to be assessed. The spatial distribution is shown in Fig. 1.

To derive the maximum amount of information from G-KB, the expectation of entropy must reach the maximum, which means Format (3) needs to reach the maximum under the constraint of

4 $$E\left[ {g_{\alpha } } \right] = \int {g_{\alpha } \left( {\chi_{map} } \right)f_{G} \left( {\chi_{map} } \right)} d\chi_{map} ,\quad \alpha = \left( {1,2, \ldots ,N_{C} } \right)$$

where $N_{C}$ is total number of constraints. $g_{\alpha }$ generally represents normalized constraints, expectation (first-order moment) constraints, variance (second-order moment) constraints or covariance (second-order mixed moment) constraints. In this paper, $g_{\alpha }$ indicates covariance of morbidity in $\left( {s,t} \right)$ and $\left( {s + h_{s} ,\,t + h_{t} } \right)$ in Anhui Province [31–33]:

5 $$\begin{aligned} g_{\alpha } & = c_{\varepsilon } \left( {h_{s} ,h_{t} } \right) = c_{1} \exp \left( { - \frac{{3h_{s} }}{{a_{{h_{s} 1}} }}} \right)\left( {1 - \frac{{3h_{t} }}{{2a_{{h_{t} 1}} }} + \frac{{h_{t}^{3} }}{{2a_{{h_{t} 1}}^{3} }}} \right) \\ & \quad + c_{2} \exp \left( { - \frac{{3h_{t} }}{{a_{{h_{t} 1}} }}} \right)\left( {1 - \frac{{3h_{s} }}{{2a_{{h_{s} 2}} }} + \frac{{h_{s}^{3} }}{{2a_{{h_{s} 2}}^{3} }}} \right) \\ \end{aligned}$$

where $$c_{1}$$ and $$c_{2}$$ are sill coefficients; $$a_{{h_{s} i}}$$ and $$a_{{h_{t} i}}$$$$\left( {i = 1,2} \right)$$ represent spatial and temporal range of spatio-temporal covariance, respectively.

According to Format (3) and Constraint (4), the maximum of priori PDF is

6 $$f_{G} \left( {\chi_{map} } \right) = \frac{1}{A}\exp \left( {\sum\limits_{\alpha = 1}^{{N_{C} }} {\mu_{\alpha } g_{\alpha } \left( {\chi_{map} } \right)} } \right)$$

where $A = \int {\exp \left( {\sum\limits_{\alpha = 1}^{{N_{C} }} {\mu_{\alpha } g_{\alpha } \left( {\chi_{map} } \right)} } \right)d} \chi_{map}$ and $\mu_{\alpha }$ is lagrange multiplier, $\mu_{\alpha }$ is given by

7 $$E\left[ {g_{\alpha } } \right] = \frac{1}{A}\int {g_{\alpha } \left( {\chi_{map} } \right)\exp \left[ {\sum\limits_{\alpha = 1}^{{N_{C} }} {\mu_{\alpha } g_{\alpha } \left( {\chi_{map} } \right)} } \right]d\chi_{map} }$$

(2) Calculating posterior PDF. Based on Bayesian conditional probability, the posterior PDF of Schistosoma at unobserved point $\chi_{k}$ in Anhui Province can be derived from the posterior PDF obtained by using G-KB in the first update phase [43, 44], as is given by

8 $$f_{K} \left( {\chi_{k} } \right) = f_{G} \left( {\chi_{k} |\chi_{data} } \right) = \frac{{f_{G} \left( {\chi_{k} ,\chi_{data} } \right)}}{{f_{G} \left( {\chi_{data} } \right)}}$$

where, $$\chi_{data} = \left[ {\chi_{hard} ,\chi_{soft} } \right]$$.

### Geographical and temporal weighted regression, GTWR

The geographically weighted regression model is a classic model for spatial heterogeneity research while GTWR is an in-depth study of geographically weighted regression model. Based on the idea of local regression, the GTWR method, considering spatial temporal heterogeneity of data, embeds the temporal data, a new dimension, into regression parameters to simultaneously measure changes of data in space and time. The processes are as follows: Firstly, according to the adjustable bandwidth criterion, observation points are determined that affect the regression point. Secondly, the weight matrix is calculated by the spatial-temporal distance between observation points and the regression point and the weight function. Finally, the regression coefficient value is calculated by the weighted least square method. The GTWR model is expressed as [15, 17, 18, 45]:

9 $$y\left( {u_{i} ,v_{i} ,t_{i} } \right) = \beta_{0} \left( {u_{i} ,v_{i} ,t_{i} } \right) + \sum\limits_{k = 1}^{d} {\beta_{k} \left( {u_{i} ,v_{i} ,t_{i} } \right)x_{ik} + \varepsilon_{i} } ,\quad i = 1,2, \ldots ,n$$

where $\left( {u_{i} ,v_{i} ,t_{i} } \right)$ is the space-time coordinate of the $i$-th sample point; $u_{i}$, $v_{i}$ and $t_{i}$ are longitude coordinates, latitude coordinates and time coordinates of the $i$-th sample point, respectively. $y(u_{i} ,v_{i} ,t_{i} )$ is the dependent variable of the $i$-th sample point, which is morbidity of Schistosoma in Anhui Province in this paper. $\left( {x_{i1} ,x_{i2} , \ldots ,x_{id} } \right),\quad i = 1,2, \ldots ,n$ is an independent variable, representing significant influencing factors. $\beta_{0} \left( {u_{i} ,v_{i} ,t_{i} } \right)$ is the constant term of the $i$-th sample point. $\beta_{k} \left( {u_{i} ,v_{i} ,t_{i} } \right)$ is the regression coefficient of the $k$-th independent variable at the $i$-th sample point. $\varepsilon_{i}$ is an independent and identically distributed error term, which is usually assumed to obey $N(0,\sigma^{2} )$ for distribution.

### Spatiotemporal Kriging, STKriging

On the ground of Kriging model (ordinary kriging model is widely applied), STKriging [7] model replaces the two-dimensional spatial variogram with the three-dimensional spatio-temporal variogram to objectively describe the proximity of environmental variables in spatial-temporal domain. STKriging theory [13, 46, 47] primarily consists of spatial-temporal variable, spatial-temporal data stationarity, spatial-temporal variogram and spatial-temporal interpolation.

Providing that $Z(s,t)$ indicates morbidity of Schistosoma in county $s$ at time $t$ (unit/ year), STKriging model assumes that the spatio-temporal process[3, 13] of schistosomiasis morbidity is composed of spatio-temporal trend $m(s,t)$ and stochastic residual $\varepsilon (s,t)$:

10 $$Z(s,t) = m(s,t) + \varepsilon (s,t)$$

where $m(s,t)$ is determined by the result $y(u,v,t)$ of GTWR model ($(u,v)$ is the coordinates of the county geographical center).

The fitting residue covariance of morbidity of Schistosoma analyzed by GTWR at $(s,t)$ and $(s + h_{s} ,t + h_{t} )$ in Anhui Province merely depends on $(h_{s} ,h_{t} )$, where $h_{s}$ is the Euclidean space distance and $h_{t}$ is temporal distance. The spatio-temporal covariance of $\varepsilon (s,t)$ is

11 $$C(h_{s} ,h_{t} ) = Cov(\varepsilon (s + h_{s} ,t + h_{t} ),\varepsilon (s,t))$$

The variogram is

12 $$\gamma \left( {h_{s} ,h_{t} } \right) = \frac{1}{2}E\left\{ {\left( {\varepsilon \left( {s + h_{s} ,t + h_{t} } \right) - \varepsilon \left( {s,t} \right)} \right)^{2} } \right\}$$

The variogram adopted in the paper is nonseparable spatio-temporal Cressie-Huang model [46, 48]:

13 $$\gamma \left( {h_{s} ,h_{t} } \right) = \left\{ \begin{gathered} 0\quad h_{t} { = }h_{s} { = 0} \hfill \\ \sigma^{2} \left( {1 - \frac{{(a\left| {h_{t} } \right| + 1)}}{{\left( {\left( {a\left| {h_{t} } \right| + 1} \right)^{2} + b^{2} \left\| {h_{s} } \right\|^{{2}} } \right)^{1.5} }}} \right) \hfill \\ + C_{0} + a_{1} \left\| {h_{s} } \right\|^{{a_{2} }} \quad {\text{other}} \hfill \\ \end{gathered} \right.$$

In the spatio-temporal variability of fitting residual $\varepsilon (s,t)$, $a$ is temporal scale parameter, $b$ is spatial scale parameter, $C_{0}$ is nugget effect, $a_{1} \left\| {h_{s} } \right\|^{{a_{2} }}$ is pure spatial variability, $a_{1} ,a_{2}$ is spatial smoothing parameter, and $\sigma^{2}$ is the variance of $\varepsilon (s,t)$.

## Abbreviations

BME: Bayesian maximum entropy
STKriging: Spatiotemporal Kriging
GTWR: Geographical and temporal weighted regression
RMSE: Root-mean-square-error
AR: Absolute residual
LSTd: Daytime land surface temperature
LSTn: Night-time land surface temperature
NDVI: The normalized difference vegetation index
MT_min_: Mean minimum temperature
MT_max_: Mean maximum temperature
AIPD: Anhui Institute of Parasitic Diseases
IHT: Indirect hemagglutination test
VIF: Variance inflation factor
